# Mukbang Viewing and Eating Behaviors Among Saudi Adults: Insights from a Cross-Sectional Study

**DOI:** 10.3390/nu17243850

**Published:** 2025-12-10

**Authors:** Nawal Alissa, Maha H. Alhussain

**Affiliations:** 1 Community Health Sciences Department, College of Applied Medical Sciences, King Saud University, P.O. Box 10219, Riyadh 11433, Saudi Arabia; 2 Department of Food Sciences and Nutrition, College of Food and Agriculture Sciences, King Saud University, P.O. Box 2460, Riyadh 11451, Saudi Arabia; mhussien@ksu.edu.sa

**Keywords:** eating behavior, mukbang, Internet, Saudi Arabia, social media, food habits

## Abstract

**Background**: Mukbang, an online trend featuring individuals eating large amounts of food while interacting with viewers, has become increasingly popular worldwide, including in Saudi Arabia. This study explored how often Saudi adults watch mukbang videos and whether viewing frequency is related to emotional, external, and restrained eating behaviors. **Methods**: An online survey was completed by 160 participants, of whom 70 had watched mukbang videos in the past 30 days and were included in the analysis. **Results**: Viewing frequency was not significantly associated with any eating behavior type. Education level showed a significant relationship with viewing habits, with participants who had higher educational attainment reporting less frequent viewing; however, this association should be interpreted cautiously, as media awareness was not directly measured. **Conclusions**: Overall, the findings provide preliminary insight into how individual characteristics may shape engagement with mukbang content and highlight the need for further research that examines additional psychological and cultural factors.

## 1. Introduction

In today’s digital world, mukbang has emerged as a popular global trend. Originating in South Korea, it features hosts eating large amounts of food while interacting with viewers [1,2]. What began on AfreecaTV in the late 2000s later expanded to platforms such as YouTube and TikTok, attracting millions of viewers worldwide, including growing audiences in the Middle East [3].

Mukbang is more than a food show; it creates a social and sensory experience that blends performance, community, and consumption. Viewers engage with vivid visuals and distinctive eating sounds, often describing the content as relaxing or entertaining [4,5]. For some, mukbang also meets emotional or social needs by providing a sense of connection or satisfaction without eating [6]. However, concerns have been raised about the psychological and behavioral effects of repeated exposure to this type of content [7].

Recent studies suggest that mukbang viewing may influence eating behavior. Continuous exposure to food-centered videos can stimulate appetite, increase cravings, and encourage overeating [8,9]. Frequent viewers have been shown to engage more often in external eating, responding to the sight or sound of food rather than internal hunger cues [10]. Yeon [11] also found that heavy mukbang watchers may display higher impulsivity and lower self-control in food choices. In addition, they are more likely to order food deliveries, eat late at night, and prefer high-calorie meals [12]. Collectively, these findings indicate that mukbang may shape viewers’ perceptions of normal eating, influencing portion size, food preferences, and dietary norms.

Mukbang creators often consume large quantities of calorie-dense foods such as fried dishes, desserts, or noodles, with little attention to nutritional value [13]. This performance of overeating may normalize excessive consumption and desensitize viewers to unhealthy eating patterns. Previous studies have shown that emotional, external, and restrained eating behaviors are linked to higher BMI and greater risk of disordered eating [14,15]. These behaviors are commonly assessed using the Dutch Eating Behavior Questionnaire (DEBQ), which measures emotional eating, restrained eating, and external eating [16].

Mukbang’s rising popularity parallels the growing engagement with social media in Saudi Arabia, where platforms such as YouTube are among the most widely used [17]. At the same time, the country continues to face increasing rates of obesity and other diet-related health problems. According to the Saudi Ministry of Health [18], unhealthy eating habits and sedentary lifestyles remain key contributors to this national burden. Given the high level of digital activity, mukbang content may influence how viewers think about food and eating, potentially shaping dietary choices and eating behaviors within the Saudi context [19].

While mukbang has become a global phenomenon, most research examining its psychological and behavioral effects comes from East Asian and Western populations. Little is known about its impact in Arab societies, where eating habits are strongly influenced by cultural and family norms. Studying this trend in the Saudi context may clarify how food-related media relates to eating behaviors and support the development of culturally appropriate health and media-awareness initiatives.

The present study examined whether the frequency of watching mukbang videos is associated with emotional, external, or restrained eating behaviors among Saudi adults. By exploring these relationships, the study aims to provide initial insight into how digital food-related media may relate to eating behaviors in this context.

## 2. Materials and Methods

### 2.1. Study Design

This cross-sectional study investigated the relationship between mukbang watching frequency and eating behaviors among the Saudi population.

### 2.2. Instrument and Measures

The survey consisted of two sections. The first section collected demographic information, including age, sex, and educational level, as well as anthropometric data (weight and height). Participants self-reported all anthropometric data online. This section also included an item assessing the frequency of mukbang video viewing during the past week. Participants were asked, “How frequently did you watch mukbang videos during the past week?” with three response options: often, sometimes, and rarely. Based on their responses, participants were categorized into three groups: the frequent-watching (FW) group (often), the moderate-watching (MW) group (sometimes), and the rare-watching (RW) group (rarely).

The second section included the Dutch Eating Behavior Questionnaire (DEBQ), originally developed by Van Strien [16], which was used to assess emotional, external, and restrained eating behaviors. An Arabic translation of the DEBQ, previously used in regional research, was administered in this study. Although no formally validated Saudi-specific version of the DEBQ currently exists, steps were taken to enhance the cultural and linguistic appropriateness of the instrument for Saudi adults. The translated items were reviewed by two bilingual researchers with expertise in nutrition and familiarity with Saudi cultural norms, and minor wording adjustments were made to ensure that the phrasing aligned with commonly understood expressions related to emotions, appetite cues, and eating habits in the Saudi context.

Internal consistency reliability for the DEBQ subscales in the current sample was acceptable, with Cronbach’s alpha values of 0.84 for emotional eating, 0.78 for external eating, and 0.81 for restrained eating.

The DEBQ is a 33-item self-report instrument that measures three types of eating behavior: (1) emotional eating (13 items; e.g., “Do you get the desire to eat when you are anxious, worried, or tense?”), (2) external eating (10 items; e.g., “If you see others eating, do you also have the desire to eat?”), and (3) restrained eating (10 items; e.g., “How often do you try not to eat between meals because you are watching your weight?”). Responses were rated on a 5-point Likert scale ranging from never (1) to very frequently (5). The mean score for each subscale was calculated by dividing the total score by the number of items, yielding possible scores from 1 to 5. Higher scores indicated greater tendencies toward restrained, emotional, or external eating.

### 2.3. Participant Recruitment and Data Collection

Recruitment was conducted using convenience sampling through social media channels, including WhatsApp, Instagram, and Snapchat. On WhatsApp, the survey link was shared in large group chats and general social circles such as university groups, community groups, and networks of friends and relatives. Instagram and Snapchat recruitment occurred through public posts and stories that allowed for broad reach. No private or individual invitations were used. Data were collected using an online Google Form survey, which took approximately 10 min to complete. Inclusion criteria required participants to be Saudi nationals aged 18 years and older, residing in Saudi Arabia, and having watched mukbang videos within the past 30 days. Individuals younger than 18 years or those who had not watched mukbang videos in the past month were excluded. Ethical approval for the study was obtained from the Research Ethics Committee at King Saud University, Riyadh, Saudi Arabia (KSU-HE-25-085). Informed consent was obtained electronically from all participants prior to participation. Data collection was conducted during February and March 2025.

### 2.4. Sample Size Calculation

The required sample size was estimated using Cochran’s formula for an unlimited population:

$$n=\frac{z^{2}\times p(1-p)}{e^{2}}$$

where *n* represents the required sample size, *z* is the z score corresponding to a 95% confidence level (1.96), *p* is the assumed population proportion (0.50), and *e* is the margin of error (0.05). Based on these parameters, the calculated minimum sample size was 384 participants. However, due to feasibility constraints and the specific nature of the study topic, mukbang viewing behavior, the number of eligible participants was limited. A total of 160 individuals completed the survey. Of these, 70 participants reported watching mukbang videos and were included in the final analysis, while 90 respondents who had not watched mukbang videos were excluded after completing only the demographic section.

### 2.5. Data Analysis

Data were screened for missing or invalid responses and analyzed using IBM SPSS Statistics version 28 (IBM Corp., Armonk, NY, USA). Descriptive statistics were presented as means and standard deviations (SD) for continuous variables and as frequencies and percentages (%) for categorical variables. The Kolmogorov–Smirnov test was used to assess the normality of continuous variables. Independent samples *t*-tests were performed to compare mean differences between mukbang viewers and non-viewers, and Pearson chi-square tests were applied to examine associations between categorical variables.

For participants who reported watching mukbang videos, Spearman rank-order correlations were conducted to examine the associations between viewing frequency, eating behavior subscale scores, and demographic variables. In addition, simple linear regression analyses were performed to determine whether mukbang viewing frequency could predict participants’ eating behavior scores and demographic characteristics. Statistical significance was set at *p* < 0.05 for all analyses.

## 3. Results

### 3.1. Demographic Characteristics of Participants

Although 160 individuals completed the survey, only the 70 participants who reported watching mukbang videos were included in the analyses related to eating behaviors, whereas the remaining 90 respondents contributed only demographic information.

Table 1 presents the demographic and anthropometric characteristics of the study participants, stratified by whether they reported watching mukbang videos. The mean age of participants was 24.7 ± 12.1 years, with no significant age difference between mukbang viewers and non-viewers. Similarly, no significant differences were observed in anthropometric variables, including weight, height, and BMI.

The majority of participants were female (66.3%), and the proportion of males and females did not differ significantly between groups. Regarding educational level, most participants held a bachelor’s degree (48.8%), followed by a high school or diploma qualification (36.3%), while a smaller proportion reported postgraduate degrees (6.9%). Education level was significantly associated with mukbang viewing (*p* = 0.04). Specifically, participants with secondary education or lower were much more likely to watch mukbang videos compared to other groups, with more than three-quarters of them reporting viewing.

### 3.2. Frequency of Mukbang Video Viewing

**Figure 1:**
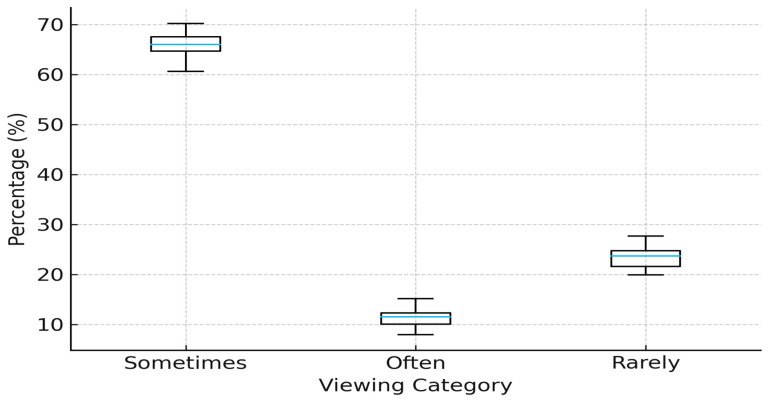
Box-and-whisker representation of mukbang viewing frequency categories among participants (*n* = 70).

Among the 70 participants who reported watching mukbang videos, the majority (*n* = 46; 65.7%) reported watching mukbang videos sometimes, followed by 22.9% (*n* = 16) who reported watching them rarely, and 11.4% (*n* = 8) who reported frequent viewing, as shown in Figure 1.

### 3.3. Average Scores of DEBQ Scales Among Mukbang Viewers

Table 2 presents the mean scores and standard deviations for each subscale of the Dutch Eating Behavior Questionnaire (DEBQ) among participants who reported watching mukbang videos (*n* = 70). Among the three eating behavior domains, external eating recorded the highest mean score (2.96 ± 0.64), followed by restrained eating (2.69 ± 0.74) and emotional eating (2.35 ± 0.87). These findings indicate that participants who watched mukbang videos tended to score higher on external eating, reflecting a greater tendency to eat in response to external food-related cues.

### 3.4. Relationship Between Mukbang Viewing Frequency and Eating Behaviors

A Spearman rank order correlation was used to examine the association between how often participants watched mukbang videos and their eating behavior scores on the DEBQ. The analysis indicated no significant associations between viewing frequency and any of the three eating behavior types: restrained, emotional, or external eating.

When considering demographic variables, there were also no significant associations between mukbang viewing frequency and participants’ age, BMI, or sex. Education level showed a weak positive trend (*rₛ* = 0.20, *p* = 0.096), suggesting that participants with higher levels of education tended to watch mukbang videos less frequently; however, this relationship did not reach statistical significance (Table 3).

Simple linear regression analyses were then conducted to further explore whether mukbang viewing frequency could predict participants’ eating behavior scores and demographic characteristics. The models for restrained eating (*R*^2^ = 0.016, *p* = 0.304 *), emotional eating (*R*^2^ = 0.039, *p* = 0.102 *), and external eating (*R*^2^ = 0.012, *p* = 0.365 *) were not statistically significant. 

Among the demographic variables, only the regression model predicting education was statistically significant (*R*^2^ = 0.056, *p* = 0.049), indicating that participants with higher educational level were less likely to watch mukbang videos frequently. Regression models for age, sex, and BMI were not significant (Table 4).

## 4. Discussion

The findings of this study indicated that mukbang viewing frequency was not significantly associated with emotional, external, or restrained eating behaviors among Saudi adults. Education level emerged as the only significant factor, with participants who had higher educational attainment reporting less frequent viewing. These results suggest that individual characteristics may play a more important role in mukbang engagement than specific eating behavior tendencies.

The results of this study are consistent with a body of research that presents mixed findings on mukbang viewing and eating behaviors. Previous studies suggest that the relationship between watching mukbang videos and eating patterns is multifaceted and influenced by several contextual factors. For instance, Anari and Eghtesadi [12] reported that among Iranian female students, the frequency of mukbang viewing was not related to emotional or restrained eating but showed a positive association with external eating, particularly when viewing time increased. Similarly, Jang and Park [20] found that among Korean children and adolescents, longer periods of mukbang viewing were linked to meal skipping, more frequent late-night snacking, and lower overall diet quality. Collectively, these findings indicate that mukbang exposure may be connected to externally driven or cue-based eating behaviors in certain populations and under specific circumstances.

In the current Saudi sample, however, viewing frequency was not significantly associated with restrained, emotional, or external eating. One explanation is that simple frequency measures may not capture the underlying processes that connect media use with eating outcomes. Kim et al. [21] showed that the effects of eating broadcast consumption depend on viewers’ motivations such as entertainment, relaxation, social companionship, or mood regulation. Individuals who watch primarily for companionship or relaxation may respond differently than those who watch for food stimulation or while already hungry. This may explain why viewing frequency alone often produces non-significant results even though some individuals report changes in appetite or craving.

Cultural context also appears to play an important role. In Saudi Arabia, social norms that emphasize family meals and hospitality may buffer against individualistic or vicarious eating prompted by online food content.

While shared family meals and hospitality are common across many cultures, certain aspects of Saudi social norms may uniquely influence how mukbang content is interpreted. In Saudi households, meals are often highly social events centered around communal eating, large portions, and an emphasis on generosity and hosting. Because mukbang also highlights abundant food and a sense of social connection, viewers in this context may relate to the content in ways that reflect these familiar cultural practices. This may help explain why some individuals find mukbang comforting, engaging, or culturally resonant, even though the behavior itself is mediated through digital platforms. By clarifying this connection, we aim to situate mukbang engagement within the broader cultural environment that shapes food-related attitudes in Saudi society.

Studies from other regions have shown that audiences interpret mukbang differently depending on cultural setting, with many Arab and East Asian viewers perceiving it primarily as a form of entertainment rather than a direct cue to eat [22]. Moreover, research on the Arabic version of the Mukbang Addiction Scale has indicated that maladaptive or compulsive engagement rather than general exposure is more strongly associated with adverse outcomes such as disordered eating and psychological distress [23]. These findings support the interpretation that ordinary viewing frequency alone does not necessarily predict unhealthy eating behaviors.

The inverse association observed between education and mukbang viewing frequency is also consistent with broader media research, which shows that higher educational attainment is linked to greater media literacy and more selective exposure to online content [21]. Education may therefore serve as a protective factor by influencing how individuals manage and regulate their media consumption. However, the small effect size observed in this study suggests that education is only one of several factors that shape mukbang engagement. Future studies should examine additional variables such as viewing motivations, social context, and parasocial relationships to better understand these patterns.

The findings of this study have several implications for public health, media literacy, and behavioral science. The absence of a significant association between mukbang viewing frequency and eating behaviors suggests that viewing frequency alone may not be a reliable indicator of vulnerability to unhealthy eating patterns. Instead of focusing only on exposure levels, interventions should take into account the psychological and situational factors that influence engagement. Understanding viewers’ motivations, emotional states, and the circumstances in which mukbang is consumed, such as whether viewing occurs during meals or in solitary settings, may provide more meaningful insight into behavioral outcomes.

The observed relationship between education and viewing habits also highlights the potential role of media literacy and digital health education in promoting healthier media engagement. Incorporating these elements into nutrition and behavioral health programs could help individuals critically evaluate food related content and become more aware of how visual cues may influence appetite, cravings, and perceptions of normal eating behavior.

Finally, the results emphasize the importance of adopting culturally grounded approaches when studying the relationship between media and eating behaviors. Eating practices are deeply shaped by cultural values and social traditions that determine how individuals interpret and respond to food related media. In the Saudi context, customs that encourage shared meals and hospitality may affect how mukbang content is perceived and experienced. Recognizing these cultural factors is essential for developing research and interventions that are contextually relevant and effective in promoting healthy behavioral outcomes.

A major strength of this study is its contribution to a relatively underexplored topic in the Saudi and Middle Eastern context, thereby expanding the cultural scope of mukbang research. The use of a validated measure of eating behaviors (the DEBQ) and the inclusion of multiple demographic and anthropometric variables enhance the methodological rigor of the findings. Moreover, the study provides one of the first quantitative examinations of mukbang viewing habits and their potential behavioral correlates in an Arab population, helping to bridge a gap in cross-cultural media and health research.

Despite these strengths, several limitations should be acknowledged. The cross-sectional design prevents causal inference, meaning it cannot determine whether mukbang viewing influences eating behaviors or whether pre-existing tendencies shape viewing preferences. It is important to note that although 160 participants completed the survey, only 70 met the inclusion criteria for the analytic sample. This reduction in sample size limits the statistical power of the study and constrains the extent to which the findings can be generalized to the broader Saudi adult population. Although the Arabic version of the DEBQ demonstrated acceptable internal consistency in our sample (Cronbach’s alpha 0.78–0.84), it has not been formally validated for Saudi populations, which may affect the cultural and linguistic precision of the measure. A full validation study tailored to the Saudi context is therefore warranted.

The reliance on self-reported data for both media consumption and eating behaviors introduces the possibility of recall bias and social desirability effects, particularly for weight-related variables and eating patterns. Moreover, the study focused only on viewing frequency as the main exposure variable. This approach does not capture other important aspects of engagement, such as viewing duration, emotional reactions while watching, or the specific types of mukbang content consumed.

While the study provides initial insight into mukbang viewing habits among Saudi adults, the findings should be interpreted with caution. The analytic sample was relatively small and drawn through convenience sampling, which limits the representativeness of the results. In addition, although the DEBQ showed acceptable reliability in our sample, it has not been formally validated for the Saudi population, which may affect the precision with which eating behaviors were measured. These factors, together with the cross-sectional nature of the study, mean that the relationships observed should be viewed as preliminary. The results cannot be generalized to the broader Saudi adult population, nor can they be used to infer causality. Instead, they offer an early indication that can guide future, more rigorous research.

Because the design is correlational, the associations observed cannot be interpreted as causal. It is not possible to determine whether watching mukbang influences eating behaviors, or whether individuals with certain eating tendencies are more likely to seek out mukbang content. For example, people who experience emotional or external eating may be drawn to mukbang for comfort, entertainment, or distraction. These alternative explanations highlight the need for longitudinal or experimental studies to better understand the direction of these relationships.

Future research should build on these findings using larger and more diverse samples to enhance generalizability. Longitudinal and experimental designs are needed to clarify the direction of associations between mukbang viewing and eating behaviors. Incorporating measures of viewing motives, media literacy, personality traits, and parasocial relationships would deepen understanding of why individuals engage with mukbang. Examining specific content types and the emotional or physiological reactions experienced during viewing may also clarify behavioral pathways. Additionally, qualitative approaches such as interviews or focus groups could capture the social and psychological meanings attached to mukbang consumption. Finally, integrating objective indicators—such as dietary records, appetite tracking, or hormonal measures—would strengthen future work by complementing self-report data and providing a more robust assessment of mukbang’s potential influence on eating behaviors.

## 5. Conclusions

This study did not find significant associations between the frequency of watching mukbang videos and emotional, external, or restrained eating behaviors among Saudi adults. Education level appeared to play a role, as individuals with higher educational attainment reported watching mukbang less frequently. However, the study did not assess media literacy or media awareness directly, so this pattern should be interpreted cautiously and considered a possible explanation rather than a confirmed mechanism. Although the findings do not indicate a direct link between viewing frequency and disordered eating, they provide preliminary insight into how cultural, educational, and motivational factors may shape patterns of media engagement within the Saudi context.

More broadly, the study highlights the importance of examining mukbang consumption through a culturally informed perspective that acknowledges psychological motives and social norms. As mukbang continues to gain popularity across different societies, understanding its potential influence on eating attitudes and behaviors remains a relevant area of research in both nutrition and media psychology. Continued investigation using diverse methodological approaches will be essential to clarify the complex and context-dependent nature of this evolving digital food phenomenon.

## Figures and Tables

**Table 1:** Descriptive Characteristics of Participants (*n* = 160).

Variable	Total (N = 160)	Watching Mukbang Videos		*p*-Value *
		Yes n = 70 (43.7%)	No n = 90 (56.3%)	
Age (year)	24.67 ± 12.1	26.19 ± 13.4	23.50 ± 10.8	0.16
Weight (kg)	67.34 ± 18.3	68.66 ± 20.0	66.28 ± 16.9	0.42
Height (cm)	165.08 ± 9.6	164.27 ± 8.9	165.71 ± 10.1	0.35
BMI (kg/m^2^)	24.4 ± 5.1	25.13 ± 5.3	23.86 ± 4.8	0.12
Sex				
Male	54 (33.8)	23 (42.6)	31 (57.4)	0.83
Female	106 (66.3)	47 (44.3)	59 (55.7)	
Education				
Secondary or lower	13 (8.1)	10 (76.9)	3 (23.1)	0.04
High School/Diploma	58 (36.3)	21 (36.2)	37 (63.8)	
Bachelor’s	78 (48.8)	33 (42.3)	45 (57.7)	
Postgraduate	11 (6.9)	6 (54.5)	5 (45.5)	

Values are presented as mean ± SD for continuous variables or frequency (%) for categorical variables. * *p*-value tested by independent *t*-test for continuous variables and Pearson’s chi-square for categorical variables, *p*-value significant < 0.05.

**Table 2:** Mean DEBQ Subscale Scores Among Mukbang Viewers (*n* = 70).

DEBQ Scales	Mean ± SD
Restrained eating	2.69 ± 0.74
Emotional eating	2.35 ± 0.87
External eating	2.96 ± 0.64

Abbreviations: DEBQ, Dutch Eating Behavior Questionnaire.

**Table 3:** Spearman Correlations Between Mukbang Viewing Frequency, Eating Behaviors, and Demographic Variables (*n* = 70).

Variable	Correlation Coefficient (ρ)	*p*
Restrained eating	0.15	0.204
Emotional eating	−0.22	0.074
External eating	−0.18	0.137
Age	0.175	0.146
BMI	0.047	0.702
Sex	0.136	0.260
Education	0.200	0.096

Note: Spearman’s rank-order correlation used for all variables.

**Table 4:** Simple Linear Regressions Predicting Eating Behaviors and Demographic Variables from Mukbang Viewing Frequency (*n* = 70).

Dependent Variable	*B*	*SE B*	β	*p*	*R* ^2^
Restrained eating	0.160	0.155	0.125	0.304	0.016
Emotional eating	−0.295	0.178	−0.197	0.102	0.039
External eating	−0.121	0.133	−0.110	0.365	0.012
Age	5.090	2.749	0.219	0.068	0.048
Sex	0.114	0.098	0.139	0.250	0.019
Education	0.347	0.173	0.237	0.049 *	0.056
BMI	0.041	1.122	0.004	0.971	0.000

Note: * indicates statistical significance at the *p* < 0.05 level.

## Data Availability

The data presented in this study are available on request from the corresponding author. The data are not publicly available due to privacy restrictions.
